# Hypoxia Antagonizes Glucose Deprivation on Interleukin 6 Expression in an Akt Dependent, but HIF-1/2α Independent Manner

**DOI:** 10.1371/journal.pone.0058662

**Published:** 2013-03-08

**Authors:** Sung Ji Choi, Ik Jae Shin, Kang-Hoon Je, Eun Kyoung Min, Eun Ji Kim, Hee-Sun Kim, Senyon Choe, Dong-Eog Kim, Dong Kun Lee

**Affiliations:** 1 Laboratory of Genome to Drug Medicine, Joint Center for Biosciences, Incheon, Korea; 2 Molecular Imaging and Neurovascular Research (MINER) Lab, Department of Neurology, Dongguk University Ilsan Hospital, Goyang, Korea; 3 Department of Neuroscience, College of Medicine, Ewha Womans University, Seoul, Korea; Rutgers University, United States of America

## Abstract

Although both glucose deprivation and hypoxia have been reported to promote cascades of biological alterations that lead to induction of inflammatory mediators, we hypothesized that glucose deprivation and hypoxia might show neutral, synergistic or antagonistic effects to each other on gene expression of inflammatory mediators depending on the regulatory components in their promoters. Gene expression of interleukin 6 (IL-6) was analyzed by real-time PCR, ELISA, or Western blot. Effects of glucose deprivation and/or hypoxia on activation of signaling pathways were analyzed by time-dependent phosphorylation patterns of signaling molecules. We demonstrate that hypoxia antagonized the effects of glucose deprivation on induction of IL-6 gene expression in microglia, macrophages, and monocytes. Hypoxia also antagonized thapsigargin-induced IL-6 gene expression. Hypoxia enhanced phosphorylation of Akt, and inhibition of Akt was able to reverse the effects of hypoxia on IL-6 gene expression. However, inhibition of HIF-1/2α did not reverse the effects of hypoxia on IL-6 gene expression. In addition, phosphorylation of p38, but not JNK, was responsible for the effects of glucose deprivation on IL-6 gene expression.

## Introduction

Glucose is the primary, if not the sole, energy substrate of the brain, and hypoglycemia in the brain causes a cerebral dysfunction ranging from mild behavioral impairment to coma [1]. Hypoglycemia occurs most commonly in diabetic patients with insulin treatment and rarely in normal subjects with prolonged fasting or in patients with hepatic failure or insulin-secreting tumors. Severe and prolonged hypoglycemia induces cell death in the brain, and studies demonstrated that mild hypoglycemia could cause changes in brain function even in the absence of neuronal death and prior to any detectable change in brain ATP concentrations [2], [3].

Glucose deprivation disrupts calcium homeostasis in the endoplasmic reticulum (ER) and activates unfolded protein response [4], [5], [6], resulting in elevation of intracellular calcium concentration ([Ca^2+^]*i*) [7]. The resultant ER stress, initiated by three ER transmembrane proteins, is an adaptive mechanism to limit cell damage. While release of calcium ion from the ER storage or influx of extracellular calcium ion raises [Ca^2+^]*i*, mitochondrial uniporter rapidly sequesters calcium ion and decreases [Ca^2+^]*i* [8]. Thapsigargin, an inhibitor of sarco/endoplasmic reticulum calcium ion ATPase, release calcium ion from the endoplasmic reticulum, raises [Ca^2+^]*i*, and activates ER stress [9]. Studies indicate that ER stress is linked to inflammatory signaling pathways, such as c-Jun N-terminal kinases (JNK) and nuclear factor κB (NFκB) pathways [10]. Activation of ER stress has been reported to increase expression of interleukin (IL)-6 [11], [12]. A recent study demonstrated that glucose deprivation, but not hypoxia or amino acid deprivation, induced IL-6 gene expression in human renal cortical cells [12].

In addition to its continuous requirement of glucose, the brain, which accounts for one fifth of total resting oxygen consumption of body [13], is very sensitive to a decrease or loss of oxygen. Cerebral hypoxia is classified by the cause of reduced supply of oxygen: hypoxic, anemic, hypemic, ischemic, and histotoxic hypoxia. Other than the common factor of a reduced supply of oxygen to the brain, each type of hypoxia provides different environments to cells affected. While ischemic hypoxia due to inadequate blood flow to the brain may cause oxygen and glucose deprivation (OGD), hypoxic, anemic, hypemic, and histotoxic hypoxia do not accompany glucose deprivation.

Reduced oxygen supply to the brain has been reported to initiate inflammatory response. Hypoxia enhanced IL-1β expression in microglia, immune cells in the brain, and murine microglial BV2 cells [14]. Ischemia-like insult, OGD followed by reoxygenation, enhanced IL-6 expression in mixed glial cells [15]. Hypoxia has also been reported to attenuate [16] or enhance expression of IL-6 [17], [18] in various types of cells. Since cerebral hypoxia may or may not accompany glucose deprivation, effects of hypoxia on expression of IL-6 need to be systematically analyzed in conjunction with the presence or absence of glucose.

Regulatory elements of IL-6 gene include interferon regulatory factor-1, AP-1, CCAAT-enhancer-binding proteins (C/EBP), and NFκB [19], [20], [21]. Mitogen-activating protein (MAP) kinases, which phosphorylate and activate AP-1, have been reported to regulate IL-6 gene expression [19]. Although numerous studies reported production of IL-6 due to microglial activation upon ischemia-like insult, it still remains unclear if IL-6 production, along with the expression other inflammatory mediators such as inducible nitric oxide synthase (iNOS), IL-1β, tumor necrosis factor (TNF)α, and matrix metalloproteinase (MMP)-9, results from hypoxia/reoxygenation or glucose deprivation. Glucose deprivation induces inflammatory mediators mainly through activation of ER stress signal pathway [12], whereas hypoxia does through activation of HIF-1α and Akt signaling pathways [22], [23]. These led us to hypothesize that glucose deprivation and hypoxia could become neutral, synergistic or antagonistic to each other on gene expression of IL-6, probably depending on the regulatory components in the promoter.

## Materials and Methods

### Reagents

Antibodies against Akt, phosphorylated Akt, p38, phosphorylated p38, extracellular signal regulated kinase (ERK)1/2, phosphorylated ERK1/2, c-Jun N-terminal kinases (JNK)1/2, phosphorylated JNK1/2, eukaryotic initiation factor 2α (eIF2α), phosphorylated eIF2α, inhibitory factor IκB and β-tubulin were purchased from Cell Signaling Technology (Danvers, MA). Akt inhibitor triciribine, HIF-1/2α inhibitor FM19G11, glycogen synthase kinase (GSK)-3β inhibitor CHIR99021, and thapsigargin were purchased from Merck.

### Cell Culture and Treatment

Characteristics of murine microglia BV2 and rat microglia HAPI cells are described in a recent review paper [24] and maintained in DMEM supplemented with 10% heat-inactivated fetal bovine serum (FBS), 100 U/ml penicillin, and 100 µg/ml streptomycin at 37°C in a humidified incubator containing 5% CO_2_. RAW264.7 macrophages were purchased from Korean Cell Line Bank (Seoul, Korea) and maintained in DMEM supplemented with 10% FBS, 100 U/ml penicillin, and 100 µg/ml streptomycin. Human monocytic cell line THP-1 cells were purchased from Korean Cell Line Bank (Seoul, Korea) and maintained in RPMI1640 supplemented with 10% heat-inactivated FBS, 100 U/ml penicillin, and 100 µg/ml streptomycin. Cells seeded onto 12 well culture plate one day before treatment were washed with PBS once and incubated in serum free DMEM or serum and glucose free DMEM in the normoxic (21% oxygen) or hypoxic (1 ∼ 10% oxygen) culture incubator for 7 h otherwise indicated. THP-1 cells were centrifuged, washed with PBS, resuspended in the medium, and incubated. A hypoxia cell culture incubator is a model SMA-30D from Aztec, Co (Fukuoka, Japan). Triciribine, CHIR99021, thapsigargin, and FM19G11 were dissolved in dimethylsulfoxide (DMSO) and diluted with culture medium just before treatment. Final concentration of DMSO in cell culture was 0.05%.

### Primary Microglial Cell Culture

Brain cortices isolated from neonatal (<P3) C57BL/6 mice were minced and incubated in 0.25% trypsin-EDTA containing DNase 1 (1 U/ml) for 10 min at 37°C. Trypsin was neutralized by adding an equal volume of complete DMEM medium, and mixed glial cells were filtered through a 40 µm cell strainer (BD Falcon). Mixed glial cells were maintained in complete DMEM media at 37°C and 5% CO_2_ for about two weeks. When the cells were confluent, primary microglial cells were harvested by mechanical agitation (200 rpm for 3 h). Primary microglial cells were plated on 24 well plates in 80% complete DMEM medium plus 20% conditioned LADMAC medium.

### Synthesis of cDNA and Quantitative Real-time Polymerase Chain Reaction (PCR)

Total RNA was extracted using TRIsure (Bioline, London, GB) according to the manufacturer’s instruction. Synthesis of cDNA was performed using GoScript reverse transcription system (Promega, Madison, USA) according to the manufacturer’s instruction. Analysis of mRNA expression was determined with quantitative real-time PCR using FastStart PCR Master Mixes, 4 pmole primers of β-actin as a reference gene and 4 pmole primers of the target genes, according to the manufacturer’s instruction. Sequences of primers for β-actin, iNOS, IL-1β, IL-6, TNFα, MMP-9 are shown in Table 1. Abundance of IL-6 mRNA in each sample was determined by the ΔCτ (cycle threshold), the difference between the Cτ values for IL-6 and β-actin. Relative ratios of IL-6 mRNA expression levels were defined as 2^−ΔΔCτ^ which reflects changes of IL-6 expression levels from cells with treatment compared to those from unstimulated (grown in serum free but glucose containing DMEM under the normoxia) cells. All experiments were performed at least 3 times with duplicates.

**Table 1:** Sequences of primers for real time-PCR.

gene	forward	reverse	species
**IL-6**	5′ACAAAGCCAGAGTCCTTCAGAG3′	5′GGTCCTTAGCCACTCCTTCTG3′	mouse
**IL-1β**	5′TCCAGGATGAGGACATGAGCA3′	5′GAACGTCACACACCAGCAGGT3′	mouse
**iNOS**	5′CTGCTGGTGGTGACAAGCACATTT3′	5′ATGTCATGAGCAAAGGCGCAGAAC3′	mouse
**MMP-9**	5′AAACCAGACCCCAGACTCCTC3′	5′GAGGACACAGTCTGACCTGAA3′	mouse
**TNF-α**	5′TCCCAGGTTCTCTTCAAGGGA3′	5′GGTGAGGAGCACGTAGTCGG3′	mouse
**β-actin**	5′CTAAGGCCAACCGTGAAAAG3′	5′ACCAGAGGCATACAGGGACA3′	mouse
**IL-6**	5′TTCCTACCCCAACTTCCAATG3′	5′ATGAGTTGGATGGTCTTGGTC3′	rat
**iNOS**	5′GGAGCAGGTTGAGGATTACTTC3′	5′TCAGAGTCTTGTGCCTTTGG3′	rat
**β-actin**	5′CAACTGGGACGATATGGAGAAG3′	5′TCTGGGTCATCTTTTCACGG3′	rat
**IL-6**	5′GTACATCCTCGACGGCATC3′	5′TTTCACCAGGCAAGTCTCC3′	human
**iNOS**	5′AAAGTTTGACCAGAGGACCC3′	5′TCCTTTGTTACCGCTTCCAC3′	human
**β-actin**	5′CGTGATGGTGGGCATGGGTC3′	5′ACGGCCAGAGGCGTACAGGG3′	human

### Preparation of Cell Lysates and Western Blotting Analysis

Cells treated as indicated were lysed by adding high salt cell lysis buffer (20 mM Tris-HCl/pH 7.5, 1 mM EDTA, 1 mM EGTA, 1% Triton X-100, 1 mg/ml leupeptin, 2.5 mM sodium pyrophosphate, 1 mM beta-glycerophosphate, 1 mM Na_3_VO_4_, 0.3 M NaCl, 0.5 mM phenylmethanesulfonyl fluoride) plus phosphatase inhibitor cocktail, and centrifuged at 12,000×*g* for 5 min at 4°C. Total cell lysates separated by SDS-PAGE were incubated with antibodies indicated in figures and processed for Western blot analyses using enhanced chemiluminescence detection kit.

### Enzyme-linked Immunosorbent Assay (ELISA)

Media from BV2 cells grown for 10 hr were saved for sandwich ELISA using Quantikine mouse IL-6 kit (R&D Systems, USA) according to the manufacturer’s instruction. Media were centrifuged to remove debris and 100 µl of clear media was incubated in a microplate pre-coated with monoclonal antibody specific for mouse IL-6 for 40 min, washed 5 times, incubated with enzyme-linked polyclonal antibody specific for mouse IL-6 for 1 hr, washed 5 times, and incubated with substrate solution for 30 min. Reactions were stopped by adding stop solution and amounts of IL-6 were determined with absorbance at 450 nm with background at 570 nm.

### Statistical Analysis

Data are presented as mean ± standard deviation (SD). Statistical comparison of data was determined using one-way analysis of variance, followed by the Dunnett post-hoc adjustment. A value of *P*<0.05 was considered significant.

## Results

### Effects of Glucose Deprivation and/or Hypoxia on IL-6 Gene Expression in Microglia, Macrophages, and Monocytes

**Figure 1:**
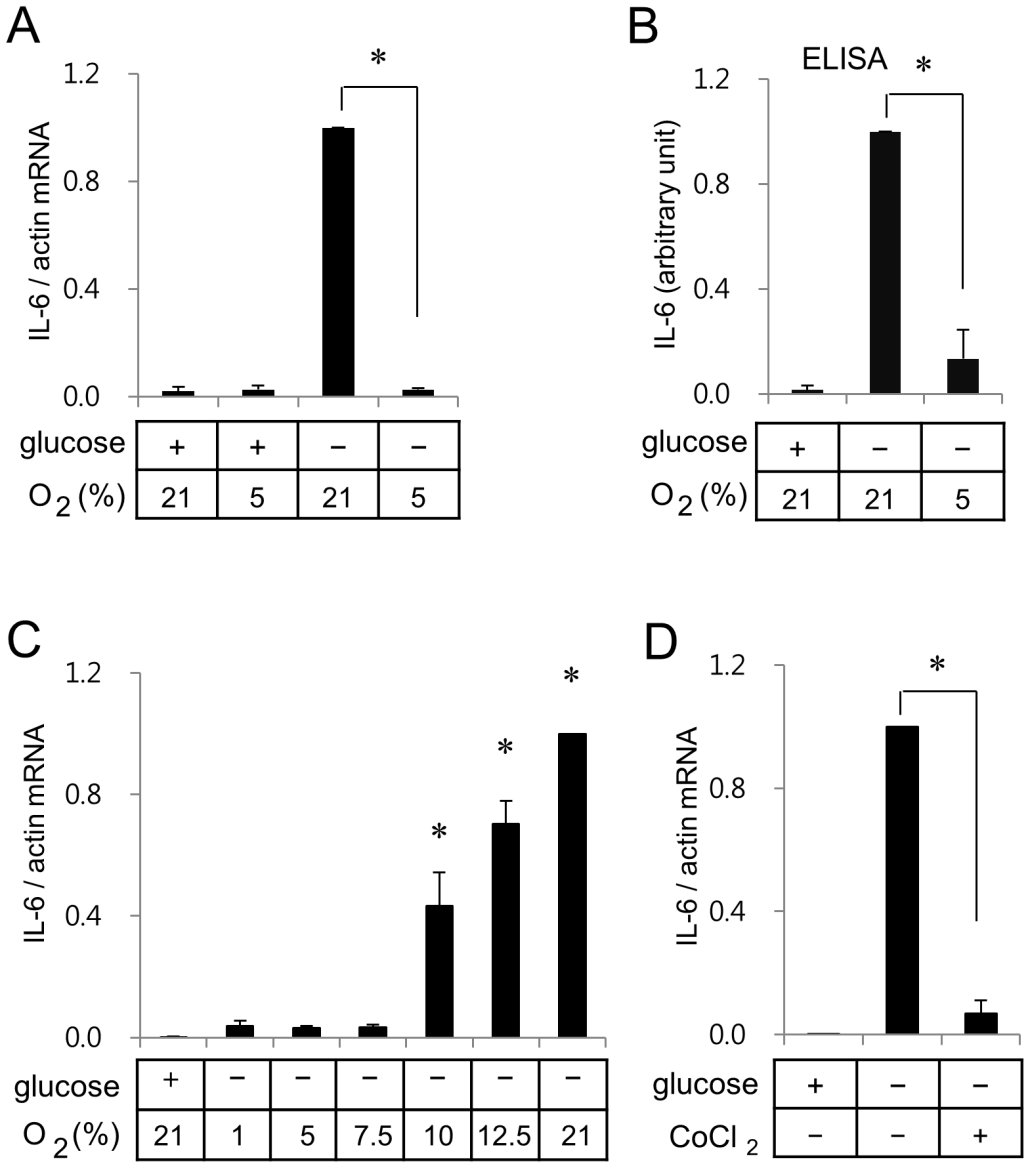
Glucose deprivation induces IL-6 gene expression, but hypoxia suppresses glucose deprivation-induced IL-6 gene expression in BV2 microglia. (A) BV2 cells were incubated for 7 h in serum free media in one of the following conditions; medium containing glucose in an incubator with 21% oxygen, medium containing glucose in a hypoxia chamber with 5% oxygen, medium without glucose in an incubator with 21% oxygen, or medium without glucose in a hypoxia chamber with 5% oxygen. Real-time RT-PCR of IL-6 was carried out with β-actin as an internal control. Ratios of IL-6 mRNA to β-actin mRNA from cells incubated in a normoxic condition without glucose were calculated as 1 in each set of experiments for statistical analysis. Results are presented as means ± SD; n (numbers of experiments performed) = 4. *p<0.01 *vs* glucose deprivation at 21% oxygen. (B) BV2 cells were incubated for 10 h and media were analyzed for ELISA as described in the Method section. Amounts of IL-6 expressed in the absence of glucose under the normoxic condition were calculated as 1 in each set of experiments for statistical analysis. Results are presented as means ± SD; n = 3. (C) BV2 cells were incubated for 7 h in a hypoxia chamber with various oxygen concentrations. (D) BV2 cells were incubated in serum free media with or without glucose. Chemical hypoxia was induced by addition of 0.2 mM CoCl_2_.

With the hypothesis that hypoxia and glucose deprivation may induce expression of inflammatory mediators by activating different signaling pathways, we assessed effects of hypoxia and/or glucose deprivation on mRNA expression patterns of IL-1β, IL-6, iNOS, TNFα, MMP-9, in BV2 murine microglia. Whereas mild hypoxia (5% oxygen) in the presence of glucose did not induce IL-6 mRNA expression, glucose deprivation under the normoxic condition (21% oxygen) markedly induced IL-6 mRNA expression (Fig. 1A). However, glucose deprivation did not induce expression of IL-1β, TNFα, and MMP-9 in BV2 cells (data not shown).

Glucose deprivation-induced IL-6 mRNA expression disappeared under the mild hypoxic condition (Fig. 1A). These findings were corroborated by ELISA measurements of the protein levels (Fig. 1B). Although IL-6 mRNA expression was up-regulated starting from 4 hr incubation, secretion of IL-6 into medium was not detected until 8 hr incubation in the glucose free medium (data not shown). We next evaluated the glucose deprivation-mediated IL-6 mRNA expression under the various concentrations of oxygen (1, 5, 7.5, 10 and 12.5%). As shown in Fig. 1C, the glucose deprivation-mediated IL-6 mRNA expression was not observed under the oxygen concentrations up to 7.5%; thereafter, it increased in an oxygen concentration dependent manner (Fig. 1C). These results suggest that oxygen depletion may have a counterbalancing effect on the glucose deprivation-induced expression of IL-6. Antagonistic effect of hypoxia on glucose deprivation-induced IL-6 expression was also observed with chemical hypoxia using cobalt chloride (Fig. 1D).

**Figure 2:**
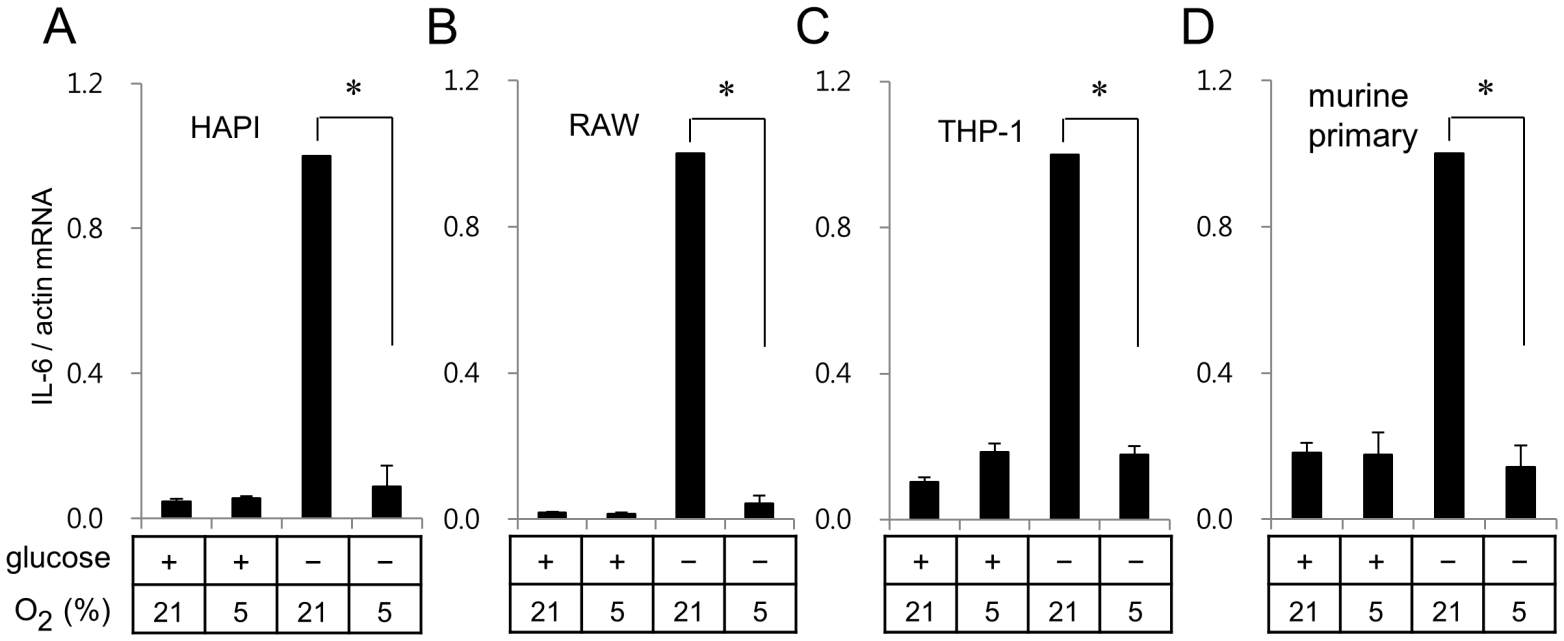
Hypoxia suppressed glucose deprivation-induced IL-6 expression in various types of cells. Rat microglial cell line HAPI cells (A), murine macrophage RAW264.7 cells (B), human monocyte THP-1 cells (C), and murine primary microglia (D) were incubated for 7 h as described in Fig. 1A. Expression of IL-6 was analyzed as described in Fig. 1A. *p<0.01 *vs* glucose deprivation at 21% oxygen.

Effects of glucose deprivation and hypoxia on IL-6 gene expression were further analyzed in murine primary microglia and different cell lines including rat microglial HAPI cells, murine macrophage RAW264.7 cells, human monocyte THP-1 cells. Glucose deprivation enhanced IL-6 mRNA expression in all cell line cells tested (Fig. 2) as well as murine primary microglia (Fig. 2). Consistent with the results obtained with BV2 cells, the mild hypoxia (5% oxygen) suppressed glucose deprivation-induced IL-6 mRNA expression in HAPI, RAW264.7, THP-1, and murine primary microglia (Fig. 2).

Glucose deprivation also showed modest induction of iNOS gene expression in various cells (Fig. S1). While hypoxia enhanced glucose deprivation-induced iNOS mRNA expression in BV2 and RAW264.7 cells, it showed no effect on the iNOS expression in HAPI and THP-1 cells (Fig. S1).

### Effects of Oxygen and/or Glucose Deprivation on Activation of the MAPK Signaling Pathway

**Figure 3:**
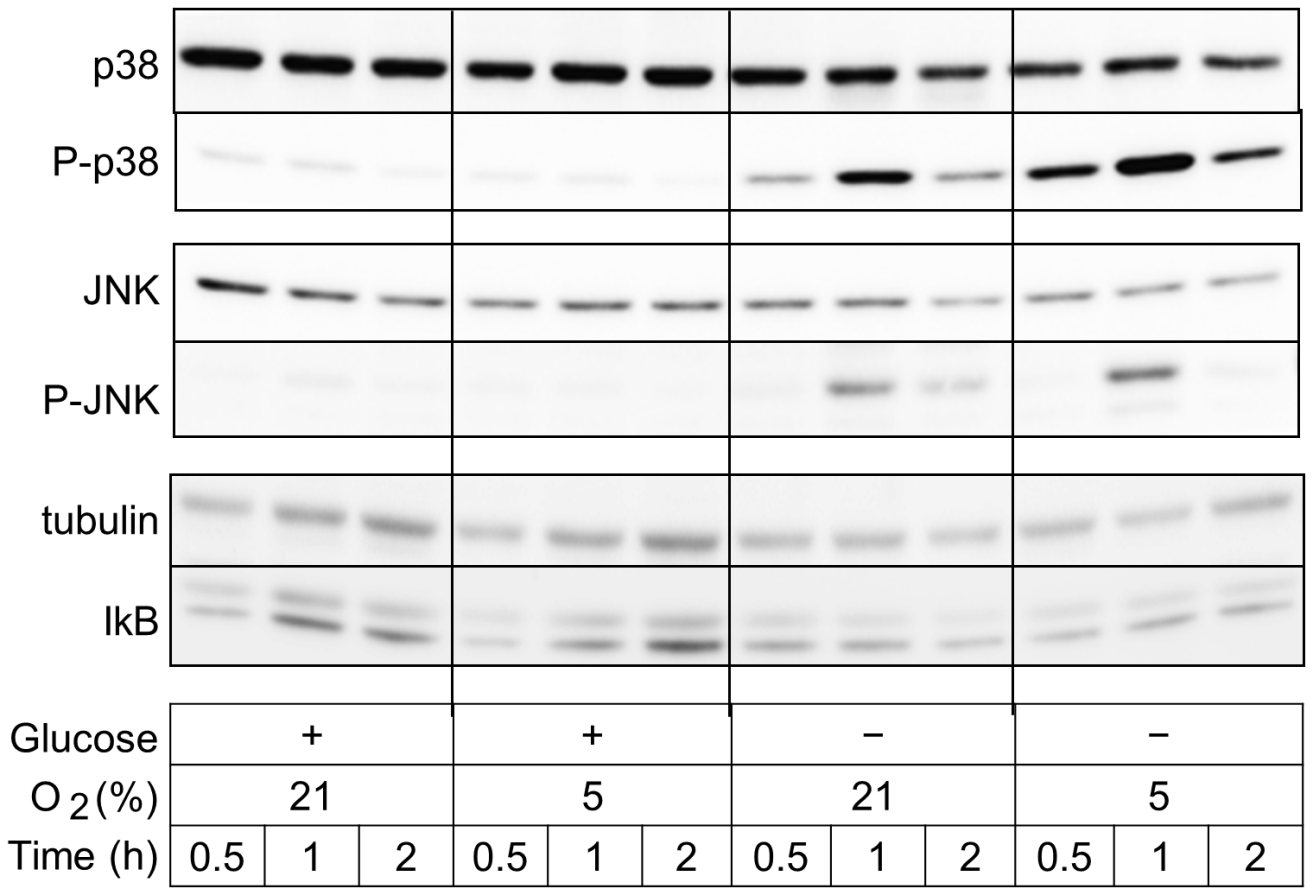
Glucose deprivation activates MAPK signaling pathway. Cells in serum-free medium were incubated for periods indicated in one of the following conditions; medium containing glucose in an incubator with 21% oxygen, medium containing glucose in a hypoxia chamber with 5% oxygen, medium without glucose in an incubator with 21% oxygen, or medium without glucose in a hypoxia chamber with 5% oxygen. Whole cell lysates separated on SDS-polyacrylamide gels were analyzed with P-p38, p38, P-JNK, JNK, β-tubulin, or IκB. Representative results out of 3 independent experiments are shown.

Since binding sites for AP-1 family transcription factors have been identified in the promoter regions of IL-6 gene [25], we analyzed effects of glucose deprivation and/or hypoxia on phosphorylation of MAP kinases, which plays a key role in activation of AP-1. Glucose deprivation, but not hypoxia alone, induced phosphorylation of both MAP kinase p38 and JNK (Fig. 3). However, glucose deprivation did not induce phosphorylation of ERK (data not shown). Hypoxia further enhanced glucose deprivation-mediated phosphorylation of p38 (Fig. 3). We also analyzed effects of glucose deprivation and/or hypoxia on the NFκB signaling pathway that has been reported to regulate IL-6 gene expression [26]. Glucose deprivation showed little effect on the relative amounts of IκB (Fig. 3). Glucose deprivation or hypoxia showed little effect on expression levels of NFκB in BV2 microglia (data not shown).

### Effect of a Mitochondrial Uniporter Agonist on the Expression of IL-6 in BV2 Murine Microglia

**Figure 4:**
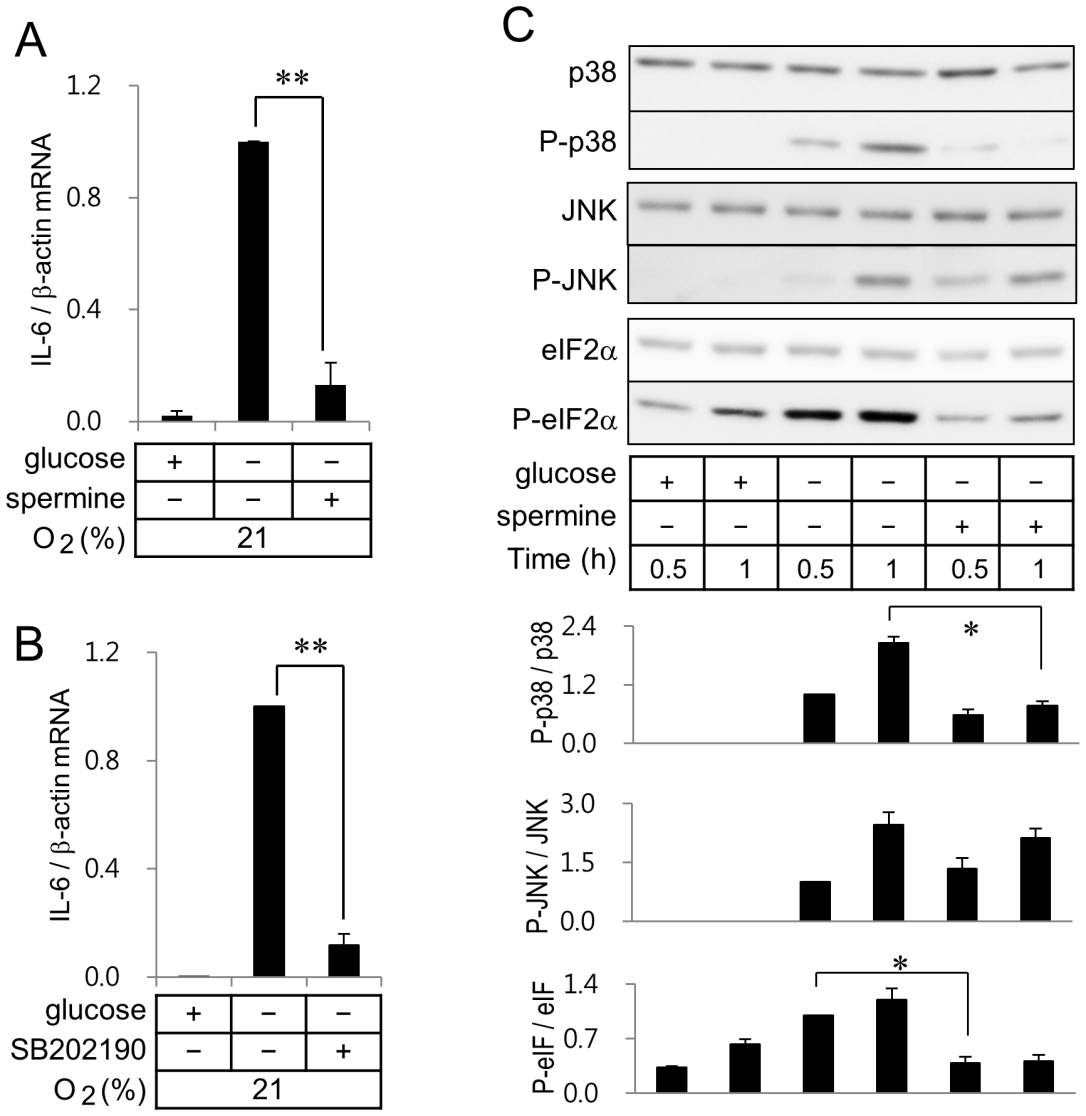
Spermine, a mitochondrial uniporter agonist, and p38 inhibitor SB202190 suppress glucose deprivation-induced IL-6 gene expression. Real-time RT-PCR of IL-6 was carried out with RNA extracted from BV2 microglia incubated in a normoxic condition with or without 1.5 mM spermine (A) or 10 µM SB202190 (B). **p<0.01 *vs* glucose deprivation without spermine or SB202190. (C) Cell lysates from cells incubated in a normoxic condition were separated on SDS-polyacrylamide gels and analyzed with antibodies against P-p38, p38, P-JNK, JNK, eIF2α, or P-eIF2α. Quantitation of protein levels of P-p38, P-JNK, P-eIF2α was calculated with those of non-phosphorylated p38, JNK, eIF2α respectively. Ratios of protein levels of interest to those of non-phosphorylated forms obtained from cells incubated in the glucose free medium without spermine for 0.5 h were calculated as 1 in each set of experiments for statistical analysis. Results are shown as means ± SD; n = 3 independent experiments. *p<0.05 *vs* glucose free medium.

We observed that spermine, a mitochondrial uniporter agonist, showed a marked suppression in glucose deprivation-induced IL-6 mRNA expression (Fig. 4A). These results demonstrated that reduction of [Ca^2+^]*i* by activation of mitochondrial uniporter suppressed IL-6 mRNA expression to an extent contributed by glucose deprivation. It was reported that p38 MAP kinase inhibitor SB202190 activated a mitochondrial uniporter [27]. The glucose deprivation-induced IL-6 mRNA expression was suppressed by addition of SB202190 (Fig. 4B).

We then evaluated effects of spermine on the glucose deprivation-mediated phosphorylation of p38 and JNK. Spermine suppressed phosphorylation of p38 that was induced by glucose deprivation. However, spermine did not show any effect on phosphorylation of JNK (Fig. 4C and D). Since spermine markedly suppressed glucose deprivation-induced IL-6 mRNA expression without suppression of JNK activity, activation of p38, but not JNK, appeared to play a key role in induction of IL-6 mRNA expression in the BV2 microglia. As reported elsewhere [12], glucose deprivation enhanced phosphorylation of eIF2α, a marker protein of ER stress. Phosphorylation of eIF2α was markedly suppressed by addition of spermine (Fig. 4C and D).

**Figure 5:**
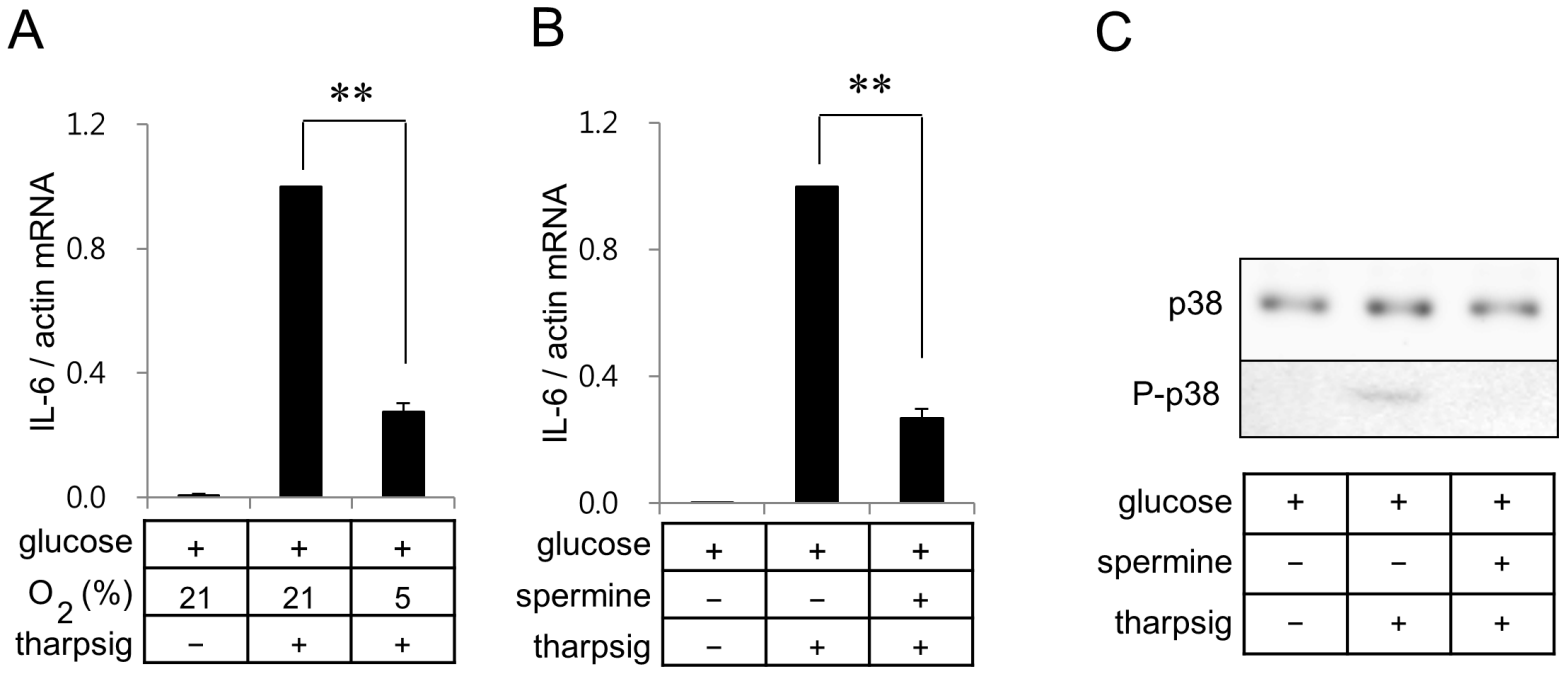
Hypoxia and spermine suppress thapsigargin-induced IL-6 gene expression in BV2 cells. Real-time RT-PCR of IL-6 was carried out with RNA extracted from BV2 microglia incubated in a glucose containing serum free DMEM under the normoxic or hypoxic conditions (A). Real-time RT-PCR of IL-6 was carried out with RNA extracted from BV2 microglia incubated in a glucose containing serum free DMEM with or without 1.5 mM spermine (B). Thapsigargin (20 nM) or spermine were added as indicated. Results are shown as means ± SD; n = 3 independent experiments. **p<0.01. (C) Whole cell lysates were separated on SDS-polyacrylamide gels and analyzed with antibodies against p38 or P-p38. Quantitation of protein levels of P-p38 were carried out as described in Fig. 4C. *p<0.05.

Since inhibition of SERCA with thapsigargin increases the cytoplasmic calcium ion concentration, we further determined if effects of hypoxia and spermine on IL-6 gene expression can be observed in BV2 cells treated with thapsigargin. Treatment of BV2 cells with 20 nM thapsigargin showed robust induction of IL-6 mRNA expression, which was suppressed by mild hypoxia (Fig. 5A) or spermine (Fig. 5B).

### Effect of GSK-3β Inhibition on IL-6 Expression in BV2 Murine Microglia

**Figure 6:**
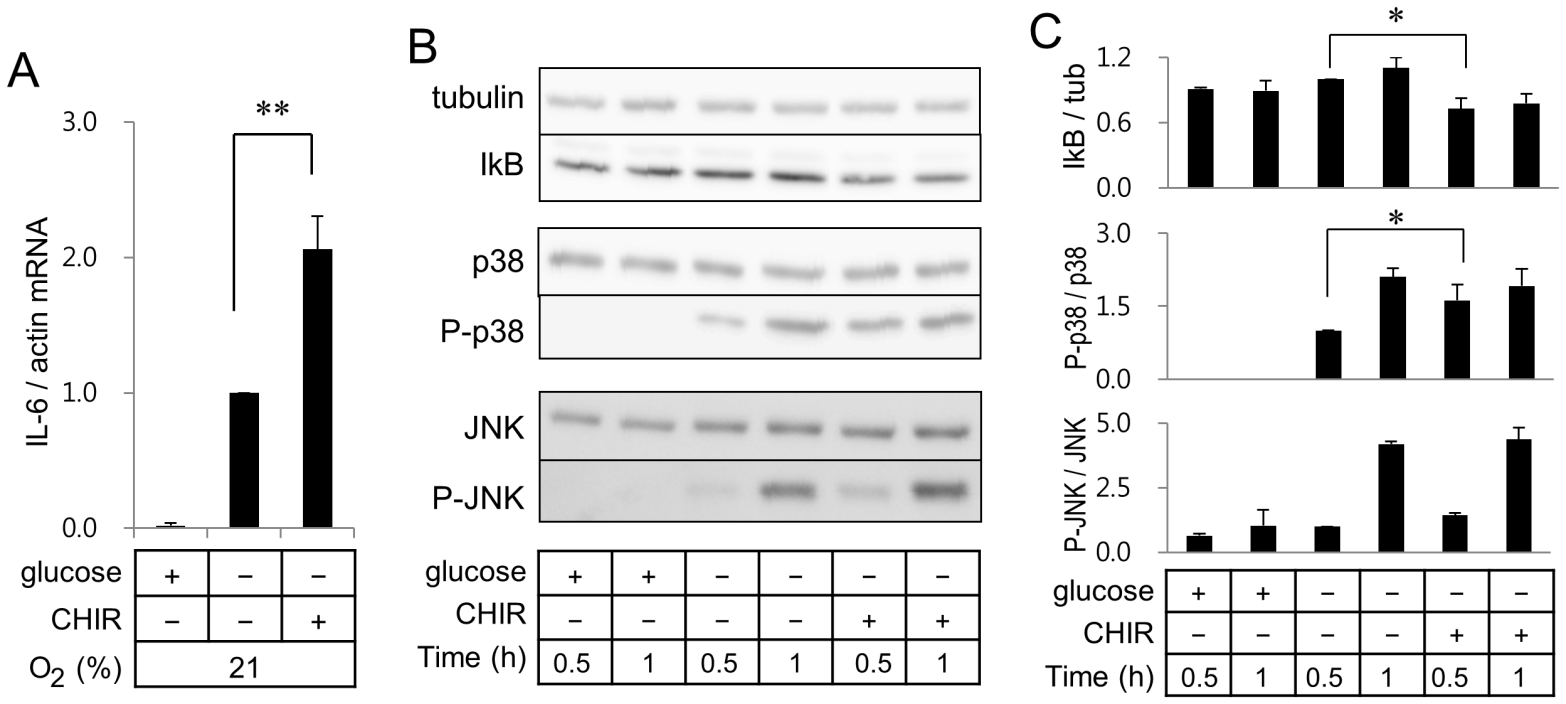
GSK-3β inhibitor enhances glucose deprivation-induced IL-6 gene expression. Real-time RT-PCR of IL-6 (A) was carried out with RNA extracted from BV2 microglia incubated in a normoxic condition with or without GSK-3 inhibitor CHIR99021. (B) Whole cell lysates from cells incubated in a normoxic condition were separated on SDS-polyacrylamide gels and analyzed with antibodies against β-tubulin, IκB, P-p38, p38, P-JNK, or JNK. (C) Quantitation of protein levels of IκB, P-p38, and P-JNK was carried out as described in Fig. 4C.

Since inhibition of GSK-3β, a multifunctional serine-threonine kinase, has been reported to suppress LPS-induced IL-6 gene expression in microglia [28], we investigated the effect of CHIR99021, a highly selective inhibitor of GSK-3β, on the ER stress-mediated IL-6 gene expression in microglia. However, CHIR99021 significantly enhanced glucose deprivation-induced IL-6 mRNA expression in BV2 cells (Fig. 6A). Experiments using actinomycin D, which inhibits de novo synthesis of RNA, indicated that up-regulation of IL-6 mRNA by CHIR99021 did not result from changes in mRNA stability (data now shown).

Inhibition of GSK-3β decreased the amount of IκB, indicating activation of the NFκB pathway by CHIR99021 (Fig. 6B and C). In addition, CHIR99021 promoted phoshporylation degree of p38 in response to glucose deprivation. However, phosphorylation of JNK was not changed by CHIR99021 (Fig. 6B and C). Results in Fig. 6 demonstrate that GSK-3β inhibition up-regulated IL-6 gene expression by enhancing activity of p38 and NFκB. LiCl, another GSK-3β inhibitor, also showed up-regulation of IL-6 and iNOS expression in BV2 microglia (data not shown).

### Effects of Akt Pathway on IL-6 Gene Expression in BV2 Murine Microglia

**Figure 7:**
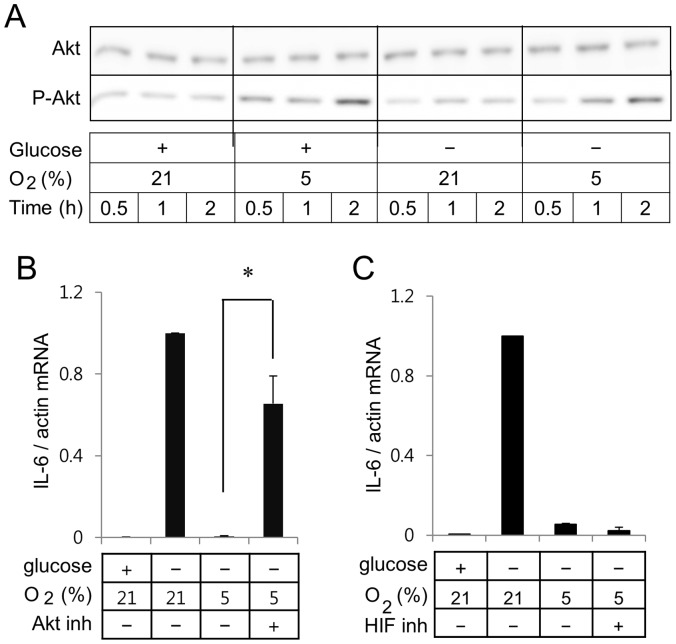
Akt inhibitor reverses effects of hypoxia on IL-6 and iNOS gene expression in BV2 microglia. (A) Cells in serum-free medium were incubated for periods indicated in one of the following conditions; media containing glucose in an incubator with 21% oxygen, media containing glucose in a hypoxia chamber with 5% oxygen, media without glucose in an incubator with 21% oxygen, or media without glucose in a hypoxia chamber with 5% oxygen. Whole cell lysates separated on SDS-polyacrylamide gels were analyzed with Akt or P-Akt. (B) Real-time RT-PCR of IL-6 was carried out with RNA extracted from BV2 cells incubated with or without Akt inhibitor. (C) Real-time RT-PCR of IL-6 was carried out with RNA extracted from BV2 cells incubated with or without 10 µM FM19G11, an inhibitor of HIF-1/2α.

Treatment of the BV2 cells in the mild hypoxia enhanced phosphorylation degree of Akt in the presence or absence of glucose (Fig. 7A). Glucose deprivation did not further enhance hypoxia-induced phosphorylation of Akt. Since hypoxia enhanced phosphorylation (activation) of Akt, we analyzed if inhibition of the Akt signaling pathway would reverse the effects of hypoxia on glucose deprivation-induced IL-6 gene expression. Addition of Triciribine, an Akt inhibitor, induced the mRNA expression of IL-6 under the hypoxic condition (Fig. 7B). However, addition of an HIF-1/2α inhibitor FM19G11 did not reverse the hypoxia-mediated suppression of glucose deprivation-induced IL-6 expression in BV2 cells (Fig. 7C).

## Discussion

The brain displays a unique requirement of continuous supply of glucose as well as high sensitivity to oxygen [1], [13]. Microglia, resident macrophages of the brain, actively monitor their environments in normal and pathogenic brains. Although ischemic insult induces microglia migration to the injury site and production of proinflammatory cytokines or mediators [29], studies of exogenious microglia transplantation [30], [31] or selective microglia ablation in ischemic animals [32] suggest neuroprotective roles of microglia [32]. Thus, microglial activation, by itself, may not be beneficial or detrimental to ischemic insult, but the effect of microglia on cerebral ischemia may be dependent on the net balance of secreted molecules from microglia.

**Figure 8:**
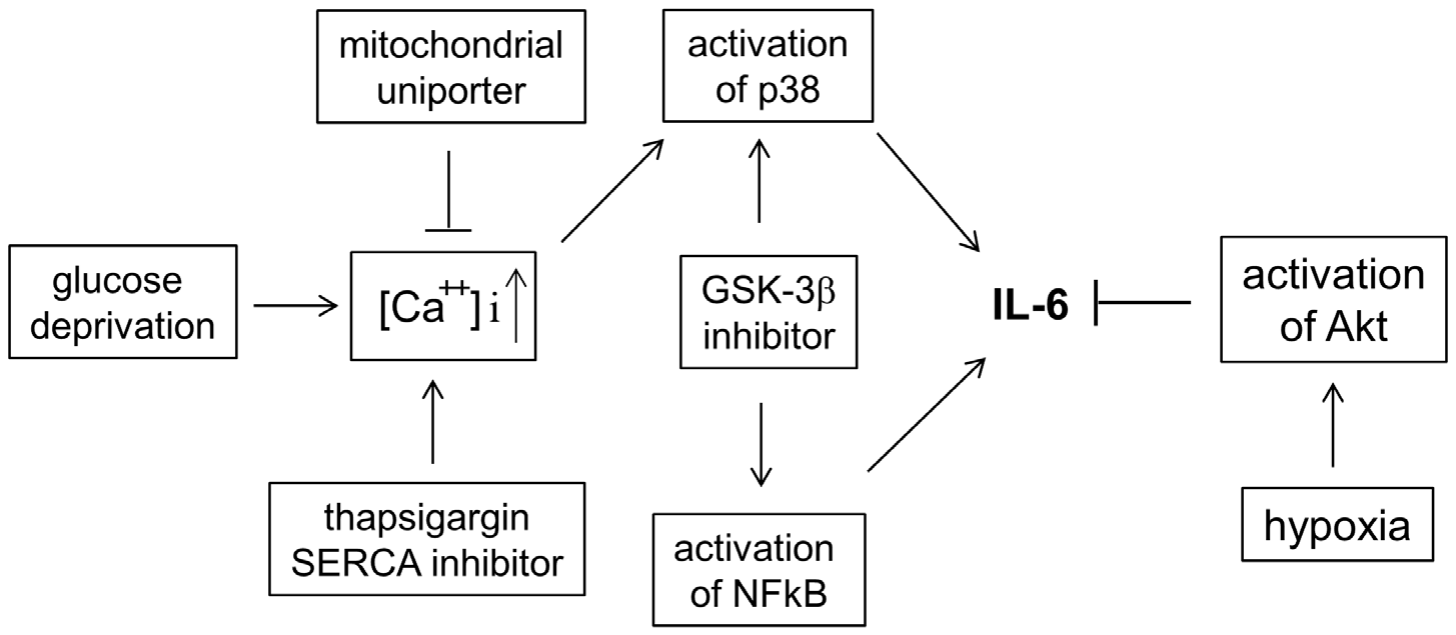
A schematic diagram explains mechanisms by which hypoxia and glucose deprivation regulates IL-6 gene expression in BV2 microglia. Glucose deprivation or SERCA inhibition increases cytoplasmic calcium ion concentration and induces IL-6 gene expression by activating NFκB and p38. Hypoxia down-regulates IL-6 gene expression by activating the Akt pathway.

Systematic analysis in the present study demonstrated effects of hypoxia and/or glucose deprivation on IL-6 gene expression in murine microglia BV2 cells (Fig. 8). There is a close link between hypoxia and inflammation; hypoxia causes inflammatory responses and inflammatory lesions become hypoxic [33]. Mice exposed to 5% oxygen for 60 min as well as humans at high altitude exhibited elevated plasma levels of IL-6 [34], although it is not clear if the elevated plasma levels resulted from induction of IL-6 gene expression or enhanced protein stability or secretion of IL-6. Results in this study using cell culture model system demonstrated that mild hypoxia suppressed glucose deprivation-induced IL-6 expression in macrophages, microglia, and monocytes. Consistent with our study, a recent study demonstrated that exposure to chronic mild hypoxia attenuated expression of monocyte chemoattractant protein-1 and IL-6 in alveolar macrophages [35].

*In vitro* studies using a cell culture model have been primarily carried out under the atmospheric condition of 21% oxygen. However, oxygen levels in various tissues are lower than the atmospheric oxygen level. While an alveolar oxygen level is ∼ 14%, normal oxygen levels in the brain range from 5 to 10% [36]. Our in vitro results that microglia experiencing ER stress induced IL-6 expression need to be confirmed in various physiologic or pathologic conditions in vivo.

Although an elevated serum level of IL-6 has been reported to be associated with poor outcomes of stroke and severe coronary atherosclerosis [37], [38], in vivo and in vitro studies demonstrated neuroprotective roles of IL-6 in brain diseases [39], [40], [41], suggesting that pleiotropic IL-6 plays multiple roles in both inflammatory and neuroprotective phases of cerebral ischemia. A systematic study of IL-6 expression in an experimental animal stroke model demonstrated that immunoreactivity of IL-6 was highest in the peri-ischemic regions and suppressed in the central infarct region [42]. Thus, it is necessary to determine if 1) microglia in the peri-ischemic regions express IL-6 and 2) IL-6 expression in the peri-ischemic regions results from ER stress. Based on our results, it is tempting to speculate that post-ischemia reperfusion and re-oxygenation might increase microglial expression of IL-6 in stroke patients with hypoglycemia.

Since glucose deprivation has been reported to induce ER stress, it is necessary to determine if ER stress stimulated by other than glucose deprivation also induces IL-6 expression in microglia. ER stress induced by an increase of cytoplasmic calcium ion concentration with thapsigargin induced a marked induction of IL-6 expression in BV2 cells. Consistent with our study, a recent study demonstrated that calcium release from ER with thapsigargin enhanced IL-6 expression in fibroblast [11]. We further demonstrated that hypoxia or spermine suppressed thapsigargin-induced IL-6 expression. Since hypoxia did not suppress phosphorylation of p38, mechanisms by which hypoxia or spermine suppress thapsigargin-induced IL-6 gene expression may be different.

GSK-3β has been reported to regulate expression of inflammatory genes by modulating activation of NFκB and transcription factor β-catenin [43]. Contrast to our results showing that CHIR99021 (Fig. 6) and LiCl (data not shown) up-regulated glucose deprivation-induced IL-6 expression or OGD-mediated iNOS expression, a recent study demonstrated that GSK-3β inhibitors suppressed NO and IL-6 production in microglia in response to lipopolysaccharide (LPS) stimulation [44]. Although LPS activates NFκB and AP-1 pathway, LPS stimulation has been also reported to regulate expression of cytokines by modulating intracellular calcium concentration and phosphorylation of cAMP-responsive element binding protein [45], [46]. Therefore, further study is necessary to determine if suppression of IL-6 and NO production in LPS-stimulated microglia by GSK-3β inhibitors does not result from regulation of the NFκB or AP-1 pathways by GSK-3β inhibitors but regulation of other pathways including the calcium pathway.

In conclusion, 1) hypoxia antagonizes to suppress glucose deprivation-induced IL-6 gene expression in macrophages, microglia, monocytes, and murine primary microglia; 2) inhibition of p38, but not JNK, by spermine or SB202190 suppresses effects of glucose deprivation or thapsigargin on IL-6 gene expression; 3) GSK-3β inhibitor enhanced IL-6 gene expression by enhancing activities of p38 and NFκB; and 4) Akt inhibitor, but not HIF-1/2α inhibitor, can reverse the effects of hypoxia on IL-6 gene expression (Fig. 8).

## Supporting Information

Figure S1 **Hypoxia synergizes with glucose deprivation to induce iNOS gene expression only in BV2 and RAW264.7 cells.** BV2, RAW264.7, and HAPI cells were incubated for 7 h as described in Fig. 1A. Real-time RT-PCR of iNOS was carried out with β-actin as an internal control. Ratios of iNOS mRNA to β-actin mRNA from cells incubated in a hypoxic condition without glucose were calculated as 1 in each set of experiments for statistical analysis. Results are presented as means ± SD; n (numbers of experiments performed) = 3.
(TIF)
