# Supplementary figures and images for: Hypoxia Antagonizes Glucose Deprivation on Interleukin 6 Expression in an Akt Dependent, but HIF-1/2α Independent Manner
Source: PLoS One. 2013 Mar 8;8(3):e58662. doi: 10.1371/journal.pone.0058662 (PMC3592797; doi:10.1371/journal.pone.0058662)

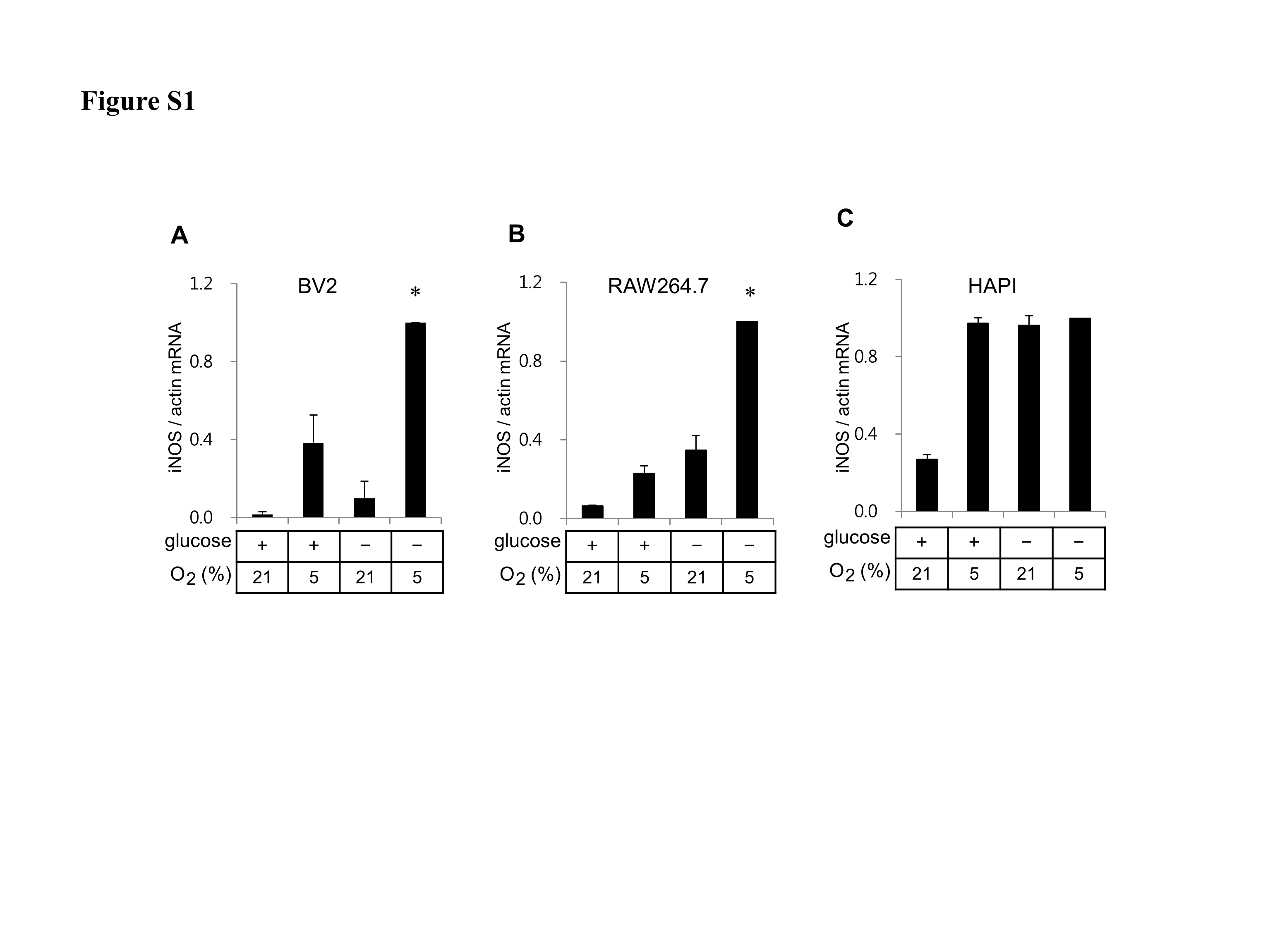

Supplement: Figure S1 — Hypoxia synergizes with glucose deprivation to induce iNOS gene expression only in BV2 and RAW264.7 cells. BV2, RAW264.7, and HAPI cells were incubated for 7 h as described in Fig. 1A. Real-time RT-PCR of iNOS was carried out with β-actin as an internal control. Ratios of iNOS mRNA to β-actin mRNA from cells incubated in a hypoxic condition without glucose were calculated as 1 in each set of experiments for statistical analysis. Results are presented as means ± SD; n (numbers of experiments performed) = 3. (TIF) [file pone.0058662.s001.tif]
